# Persistent Atrial Fibrillation Is Associated with Worse Prognosis Than Paroxysmal Atrial Fibrillation in Acute Cerebral Infarction

**DOI:** 10.5402/2012/650915

**Published:** 2012-09-27

**Authors:** Halvor Naess, Ulrike Waje-Andreassen, Lars Thomassen

**Affiliations:** Department of Neurology, Haukeland University Hospital, 5021 Bergen, Norway

## Abstract

*Background and Purpose*. We hypothesized that patients with persistent atrial fibrillation (AF) suffer from more severe cerebral infarction than patients with paroxysmal AF due to differences in clot structure and volume. *Methods*. This study includes consecutive patients with acute cerebral infarction and persistent or paroxysmal AF documented by ECG any time prior to stroke onset. The National Institute of Health Stroke Scale (NIHSS) was used to assess stroke severity on admission. Short-term outcome was determined by the modified Rankin scale (mRS) score, Barthel index, and NIHSS score 7 days after stroke onset. Risk factors were registered on admission. Eligible patients were treated with thrombolysis. *Results*. In total, 141 (52%) patients had paroxysmal AF, and 129 (48%) patients had persistent AF. NIHSS score on admission, mRS score at day 7, and mortality were significantly higher among patients with persistent AF. Thrombolysis was less effective in patients with persistent AF. *Conclusions*. Our study shows that patients with persistent AF and acute cerebral infarction have poorer short-term outcome than patients with paroxysmal AF. Differences in clot structure or clot volume may explain this.

## 1. Introduction

Both persistent and paroxysmal atrial fibrillation (AF) are frequent causes of cerebral infarction. Risk of first-ever and recurrent cerebral infarction is thought to be similar in both persistent and paroxysmal AF [1–3]. Anticoagulation is the definite treatment of choice as to both primary and secondary preventive treatment in patients with AF [4]. Further, it has been shown that short-term outcome after cerebral infarction is better among patients on prior warfarin [5].

Atrial fibrillation causes cardiac embolism due to formation of thrombus in the left atrial auriculum [6, 7]. Thrombus formation is thought to be generated by stasis, endothelial dysfunction, and hypercoagulable state [8, 9]. The pathophysiology of thrombus formation may differ between patients with paroxysmal and persistent AF. One might speculate that shorter duration of AF in patients with paroxysmal AF is associated with smaller clots and clots more liable to early recanalization than in patients with persistent AF. Clots in persistent AF may be larger due to larger left atrial auriculum. Furthermore, clots in persistent AF may possibly be more organized and consolidated and therefore less prone to early recanalization. Studies on stroke patients rarely differentiate between patients with paroxysmal and persistent AF. We hypothesized that persistent AF is associated with worse short-term prognosis than paroxysmal AF both regarding functional outcome and survival in patients with acute cerebral infarction. 

## 2. Methods

All consecutive patients with acute cerebral infarction (the index stroke) admitted to the Stroke Unit, Department of Neurology, Haukeland University Hospital, between February 2006 and February 2011, were prospectively registered in a database (The Bergen NORSTROKE Registry). Cerebral infarction was defined in accordance with the Baltimore-Washington Cooperative Young Stroke Study Criteria comprising neurological deficits lasting more than 24 hours because of ischemic lesions or transient ischemic attacks where CT or MRI showed infarctions related to the clinical findings [10]. Eligible patients were treated with intravenous thrombolysis within 4.5 hours of stroke onset. The patients included in this study had persistent or paroxysmal AF documented by ECG any time prior to stroke onset. Paroxysmal AF was defined as at least two episodes with duration less than 7 days. Persistent AF was defined as AF lasting longer than 7 days. Patients with AF diagnosed for the first time after stroke onset were excluded. 

The National Institute of Health Stroke Scale (NIHSS) was used to assess stroke severity on admission and day 7. Short-term outcome was defined by the modified Rankin scale (mRS) score and the Barthel index (BI) on day 7 or at discharge, if discharged earlier. 

Current smoking was defined as smoking at least one cigarette per day. Diabetes mellitus was considered present if the patient was on glucose-lowering diet or medication. Hypertension, angina pectoris, myocardial infarction, and peripheral artery disease were considered present if diagnosed by a physician any time before stroke onset. Aetiology was determined by the Trial of Org 10172 in Acute Stroke Treatment classification (TOAST) and classified as large-artery atherosclerosis, cardioembolism, small vessel disease, other, and unknown [11]. Clinical classification was based on the OCSP scale which includes lacunar syndrome (LACS), partial anterior circulation syndrome (PACS), total anterior circulation syndrome (TACS), and posterior circulation syndrome (POCS) [12].

Isolated acute ischemic lesions on CT or MRI were defined as lacunar infarctions (LI) if <1.5 cm and located subcortical or in the brainstem [13]. All other acute ischemic lesions were defined as nonelacunar infarction (NLI). NLI comprised subcortical and brainstem infarction ≥1.5 cm, cortical infarction, mixed cortical and subcortical infarction and cerebellar infarction. 

On admission, blood sample analyses included D-dimer, troponin, cholesterol, and fibrinogen. ECG was obtained on admission.

### 2.1. Statistics

Student's *t*-test, Mann-Whitney's test, chi-square test, and Fisher's exact test were performed when appropriate. Logistic regression and linear regression analyses were performed as detailed in the results. STATA 11.0 was used for analyses.

## 3. Results

In total, 1528 patients were admitted because of cerebral infarction: 661 (43.3%) females and 867 (56.7%) males. Prior persistent AF was known among 129 (8.4%) patients, and paroxysmal AF was known among 141 (9.2%) patients. 

Table 1 shows demographics of patients with persistent and paroxysmal AF. Patients with persistent AF were significantly more often female, older, and had more often other heart diseases. NIHSS score on admission was higher among patients with persistent AF (*P* = .001). Functional scores at day 7 (or on discharge, if discharged earlier) were worse among patients with persistent AF both regarding mRS (*P* < .001) and BI (*P* < .001). Among patients treated with thrombolysis, improvement of NIHSS score during the first week was significantly higher among patients with paroxysmal AF. Mortality within one week of stroke onset was higher among patients with persistent AF (*P* = .002). 

Logistic regression showed that persistent AF was significantly associated with high age, high NIHSS score on admission, diabetes mellitus, and prior myocardial infarction (Table 2). 

Linear regression showed that high NIHSS score on admission was significantly associated with persistent AF and sex, but not age (Table 3). Linear regression with BI day 7 (or on discharge if discharged earlier) showed that low functional outcome was associated with high age and persistent AF when adjusted for NIHSS score on admission (Table 4). Including independent variables on warfarin, thrombolysis and time to admission did not change this association. Linear regression analyses with change in NIHSS score during the first week showed significant improvement in patients with paroxysmal AF treated with intravenous thrombolysis (partial correlation = .3; *P* = .03), but not in patients with persisting AF (partial correlation = .01; *P* = 1.0) after adjusting for sex and age compared to patients not treated with thrombolysis. Logistic regression showed that mortality within 7 days of stroke onset was not associated with type of atrial fibrillation (paroxysmal versus persistant atrial fibrillation) (*P* = .12) when adjusting for sex, age, and NIHSS score on admittance.

## 4. Discussion

In support of our hypothesis, we found that patients with persistent atrial fibrillation had significantly worse short-term outcome both as to functional outcome and survival. This may partly be explained by our findings that patients with persistent atrial fibrillation had significantly more severe neurological deficits on admission. In addition, the patients with persistent atrial fibrillation had less functional improvement during the first week after stroke onset even when adjusting for the severity of neurological deficit on admission. 

More severe neurological deficits on admission suggest larger blood clots or delayed recanalization prior to admission among patients with persistent atrial fibrillation compared to patients with paroxysmal atrial fibrillation. Slow improvement after admission suggests more delayed recanalization in patients with persistent atrial fibrillation than in patients with paroxysmal atrial fibrillation. 

There are at least two possible explanations for our findings. The structure of clots in patients with persistent atrial fibrillation may differ from clots in patients with paroxysmal atrial fibrillation. Clots in paroxysmal atrial fibrillation may more often be of recent origin. This may lead to more rapid recanalization due to less organization of the clot and therefore better short-term outcome that is consistent with our findings. In support, our finding that thrombolysis was more effective in patients with paroxysmal atrial fibrillation than in patients with persistent atrial fibrillation is consistent with younger and less organized clots in paroxysmal atrial fibrillation. Patients with persistent atrial fibrillation have larger left atrium [2] than patients with paroxysmal atrial fibrillation. This may be an important cause for large thrombus formation and more severe neurological deficits in the early phase of acute cerebral infarction among patients with persistent atrial fibrillation.

Our findings have possible therapeutic implications. Because short-term prognosis was worse, and intravenous thrombolysis was less effective in patients with persistent atrial fibrillation, eligible patients with persistent atrial fibrillation may be especially suitable for intra-arterial embolectomy. 

Although death by day 7 was associated with persistent atrial fibrillation on univariate analysis, this association disappeared on multivariate analysis after adjusting for age and severity of the stroke on admission. However, it is possible that this was due to few patients dying within one week. 

The relative frequency of paroxysmal atrial fibrillation was high in our study compared to other studies. One study reported 43% with paroxysmal atrial fibrillation and 57% with persistent atrial fibrillation [2]. The ACTIVE W trial included 18% with paroxysmal atrial fibrillation and 82% with persistent atrial fibrillation [3]. The SPAF trials included 23% with paroxysmal atrial fibrillation and 77% with persistent atrial fibrillation [14]. These studies included both patients with and without stroke. Another study exclusively including patients with acute stroke reported 6% with paroxysmal atrial fibrillation and 94% with persistent atrial fibrillation [15]. The different frequencies can partly be explained by different definitions of paroxysmal atrial fibrillation and different study populations. A restrictive definition of paroxysmal atrial fibrillation is perhaps advisable in patients who have not suffered from thromboembolic complications. However, because oral anticoagulation is highly effective when it comes to prevention of cerebral infarction, a restrictive definition of paroxysmal atrial fibrillation may cause too low use of oral anticoagulation in patients who have already suffered from cerebral infarction. Most studies on stroke and atrial fibrillation do not distinguish between paroxysmal and permanent atrial fibrillation. 

A weakness of our study was that systematic transcranial Doppler examinations were not performed. We therefore have no data on time to recanalization. 

In conclusion, our study shows that patients with persistent atrial fibrillation and acute cerebral infarction have poorer short-term outcome than patients with paroxysmal atrial fibrillation. Differences in clot structure or clot volume may explain this.

## Figures and Tables

**Table 1:** Comparison between patients with paroxysmal or persistent atrial fibrillation.

	Paroxysmal atrial fibrillation	Persistent atrial fibrillation	*P*
Total	141	129	
Age, mean (standard deviation (SD))	76.5 (10.2)	82.9 (8.5)	<.001
NIHSS on admittance median (interquartile range (IQR))	5 (1–11)	9 (3–17)	.001
MRS median (IQR)	2 (1–4)	4 (2–5)	<.001
Barthel index median (IQR)	100 (60–100)	70 (15–100)	<.001
NIHSS day 7 median (IQR)	3 (0–8)	4 (1–17)	.009
Systolic blood pressure mean (SD)	163 (31)	159 (31)	.28
Body temperature mean (SD)	36.7 (.7)	36.7 (.7)	.66
Glucose mean (SD)	6.4 (2.0)	7.3 (2.2)	.001
CRP mean (SD)	13 (27)	16 (28)	.38
D-dimer	2.4 (4.2)	2.5 (3.7)	.82
Fibrinogen	3.8 (1.0)	4.1 (1.0)	.08
Cholesterol	5.1 (1.2)	5.0 (1.2)	.41
INR among patients on warfarin mean (SD)	2.3 (.9)	1.8 (.6)	.002
NIHSS score change and thrombolysis	5.6 (5.5)	−.4 (10.9)	.06

	*N* (%)	*N* (%)	

Females	58 (41)	78 (60)	.002
Prior cerebral infarction	25 (18)	29 (22)	.36
Myocardial infarction	38 (27)	22 (17)	.05
Other heart disease	32 (23)	48 (38)	.01
Hypertension	91 (65)	82 (64)	.81
Diabetes mellitus	19 (14)	29 (23)	.06
Smoking	19 (14)	13 (11)	.44
Prior depression	18 (18)	23 (34)	.02
Pior warfarin	36 (26)	48 (37)	.04
Embolic (MRI)	84 (88)	51 (84)	.49
Lacunar	12 (13)	10 (16)	
LACS*¹*	21 (15)	20 (16)	.09
TACS*¹*	33 (24)	47 (36)	
PACS*¹*	67 (48)	45 (35)	
POCS*¹*	19 (14)	17 (13)	
Mortality day 7	2 (1.4)	13 (10)	.002
Pathologic troponin	27 (25)	36 (34)	.18

^1^Clinical classification according to the OCSP scale.

**Table 2:** Logistic regression with paroxysmal versus persistent atrial fibrillation as dependent variable.

	Odds ratio	Confidence interval	*P*
Sex	.90	.50–1.6	.71
Age	.93	.90–.96	<.001
NIHSS score on admittance	.96	.93–.99	.01
Diabetes mellitus	.47	.23–.96	.04
Myocardial infarction	2.2	1.1–4.3	.02

**Table 3:** NIHSS score on admission as dependent variable.

	Partial correlation	*P*
Sex	.16	.01
Age	.06	.33
Paroxysmal versus persistent atrial fibrillation	−.15	.02

**Table 4:** Linear regression with Barthel index as dependent variable.

	Partial correlation	*P*
Sex	.07	.27
Age	−.23	<.001
NIHSS score on admittance	−.67	<.001
Persistent versus paroxysmal atrial fibrillation	.14	.03
